# A Rare Case of Thymic Rosai-Dorfman Disease Mimicking Malignancy on ^18^F-FDG PET/CT

**DOI:** 10.3390/tomography8060237

**Published:** 2022-11-28

**Authors:** Tongtong Jia, Bin Zhang, Xiaoyi Zhang, Xin Xu, Shibiao Sang, Shengming Deng

**Affiliations:** 1 Department of Nuclear Medicine, The First Affiliated Hospital of Soochow University, Suzhou 215006, China; 2 Department of Nuclear Medicine, Changshu No. 2 People’s Hospital, Suzhou 215501, China; 3 NHC Key Laboratory of Nuclear Technology Medical Transformation, Mianyang Central Hospital, School of Medicine, University of Electronic Science and Technology of China, Mianyang 621000, China; 4 State Key Laboratory of Radiation Medicine and Protection, Soochow University, Suzhou 215123, China

**Keywords:** Rosai-Dorfman disease, sinus histiocytosis with massive lymphadenopathy, histiocytosis, ^18^F-FDG PET/CT

## Abstract

Background Rosai-Dorfman disease (RDD), the massive lymphadenopathy characterized by the proliferation of sinus histiocytosis, is a relatively idiopathic benign disease with unknown etiology. We reported a rare case of thymic RDD detected by ^18^F-FDG PET/CT. A 23-year-old man with right-sided chest pain underwent ^18^F-FDG PET/CT scan, showing increased ^18^F-FDG uptake in an anterior mediastinal mass corresponding to a thymic lesion at an enhanced CT scan. The patient was referred to surgery with the clinical suspicion of thymic malignancy. The histological examination and immunohistochemical results confirmed RDD. Conclusions This was the first case report of RDD isolated to the thymus and initially presented with chest pain. Moreover, there was no characteristic painless neck lymphadenopathy at any stage of the disease course. Thus, for young patients with thymus mass, RDD should be considered a rare but possible diagnosis.

## 1. Background

The diagnosis of Rosai-Dorfman disease (RDD) was first described in 1969 and classified as a non-Langerhans cell histiocytosis. RDD is a rare benign lymphoproliferative disease of an unknown cause [1,2,3,4]. The disease is characterized by painless massive cervical lymphadenopathy associated with fever, weight loss, and night sweats in most patients^3^. Lymph nodes of the mediastinum, abdominal cavity, and pelvic cavity may also be involved^4^. Studies have shown that over 40% of cases involve extranodal sites, including the nasal cavity (11%), orbital tissue (11%), skin (10%), bone (5–10%), central nervous system (5%), lung, and salivary glands [5,6,7]. However, it is very rare to involve only an extranodal site, especially isolated thymic RDD [8,9,10]. Therefore, a thymic mass is initially diagnosed as thymic malignancy or lymphoma according to the local infiltration in clinical practice. Immunohistochemistry (IHC) remains the primary approach for the diagnosis of RDD, especially when the characteristic histiocytes show positive staining of S-100 protein, weak positive staining of CD68 protein, and negative staining of CD1a. However, ^18^F-FDG PET/CT, which sensitively detects metabolically active lesions, may not specifically distinguish benign and malignant thymic lymphoproliferative disorders. Ruth Lim et al. have found that RDD lesions exhibit higher ^18^F-FDG uptake due to increased glucose metabolism of the proliferating histiocytes and greater infiltration of inflammatory cells than tumor cells [4,10]. In the present work, we reported a rare case of thymic RDD that was difficult to distinguish from malignant lesions.

## 2. Case Presentation

**Figure 1:**
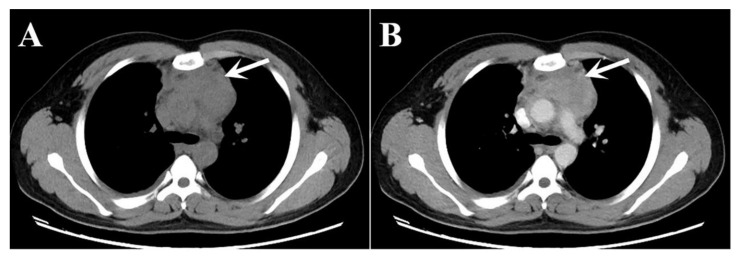
(**A**) Plain CT image; and (**B**) enhanced CT image present an abnormal soft tissue lesion in the anterior mediastinum of a 23-year-old young man (white arrows).

**Figure 2:**
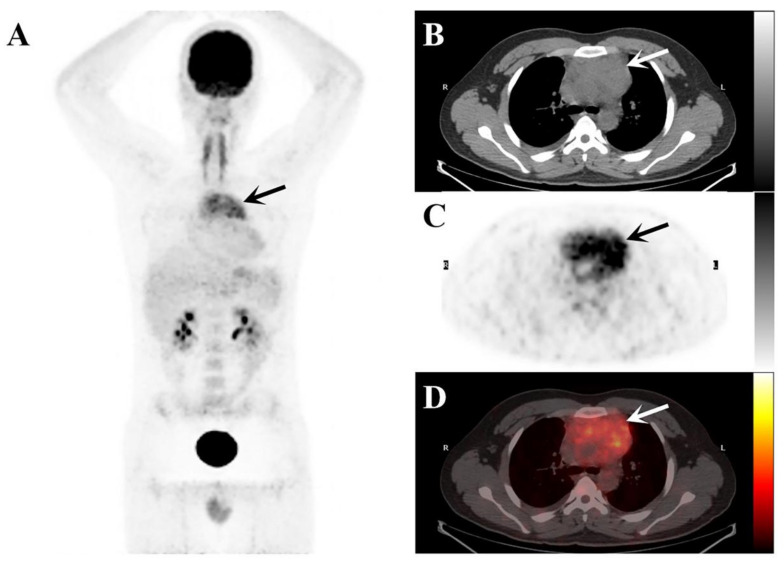
The maximum intensity projection (MIP) image of the ^18^F-FDG PET/CT (**A**) demonstrates elevated ^18^F-FDG uptake in the mediastinal region (black arrows). The axial images ((**B**), plain CT; (**C**), PET, and (**D**), fusion PET/CT) demonstrate a soft tissue mass in the anterior mediastinum (arrows) surrounding the ascending aorta.

A 23-year-old man presented with a 5-day history of right-sided chest pain. The physical examination displayed no obvious abnormalities. His laboratory tests revealed increased C-reactive protein (15.36 mg/L; normal range: 0–3 mg/L) and hyperglobulinemia (40.7 mg/L; normal range: 20–40 mg/L). The chest CT (Figure 1) and ^18^F-PET/CT (Figure 2) were used to assess the locoregional extent and clinical stage of the disease, respectively. CT examination showed a soft tissue mass in the anterior mediastinum measuring 8.5 × 5.0 cm² approximately with unclear boundaries. The plain CT value was 40 HU, and the enhanced CT value was 87 HU. This patient was initially diagnosed with lymphoma or thymoma by experienced radiologists. Subsequently, PET/CT showed abnormally high FDG uptake (SUV_max_: 8.96) in this lesion. No other abnormal ^18^F-FDG-avid lesion was observed.

**Figure 3:**
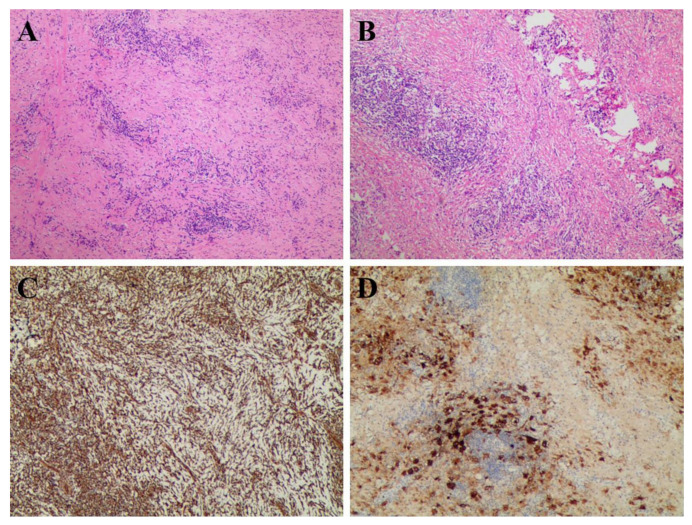
The pathology slides from the resection of the anterior mediastinal mass. (**A**,**B**) H & E staining; (**C**,**D**) IHC staining.

After imaging evaluation and a series of routine preoperative tests, the patient successfully underwent midline open resection of the mediastinal tumor. During the operation, the anterior mediastinal tumor was found to invade the pericardium, aortic arch, and superior vena cava, enveloping the left and right innominate veins. By hematoxylin-eosin (H & E) staining and IHC staining, pathologists observed biopsied sections under microscopes and reported four diagnostic slides to determine the nature of the lesion (Figure 3). Figure 3A (H & E, ×40) reveals a mixed infiltration of inflammatory cells into proliferating fibrous tissue, including histiocytes, plasma cells, and lymphocytes. At higher magnification (Figure 3B, H & E, ×100), we found the phagocytosis of inflammatory cells into the cytoplasm of histiocytes, which is called “emperipolesis”. IHC images showed that the proliferating fibrous tissue and histiocytic cells were positive for vimentin (Figure 3C). Moreover, IHC staining was positive for S-100 protein (most histiocytes), CD68 (scattered cells), and IgG4 (individual cells) but negative for CD1a (Figure 3D). These pathological findings were consistent with the clinical diagnosis of RDD.

## 3. Discussion

RDD is known as sinus histiocytosis with massive lymphadenopathy (SHML), and it is characterized by the proliferation of non-Langerhans histiocytes, demonstrating S100+, CD68+, CD1a-, and emperipolesis [11]. The typical symptoms are painless massive bilateral cervical lymph nodes with fever, neutrophil elevation, elevated erythrocyte sedimentation rate (ESR), hyperglobulinemia, and other clinical symptoms. Similar symptoms were also found in our current patient. However, RDD is not limited to lymph nodes, and extranodal organ involvements, including the skin, upper respiratory tract, bone, and so on, occur in about 40% of cases [12,13]. It is rare that the dissemination of RDD is only restricted to the thymus. Moreover, due to its unique disease location, with an unclear boundary with the ascending aorta, the chief complaint of the patient was chest pain.

Combined anatomical localization and functional metabolism, PET/CT is often used for disease diagnosis and staging evaluation. In this case, the soft tissue mass in the anterior mediastinum showed FDG avid, presumably because of histiocytosis and surrounding inflammation [14,15]. Primary thymic RDD resembles many diseases in appearance, FDG avidity, and distribution [16]. It has a wide range of differential diagnoses, including thymoma, lymphoma, infectious disease, metastatic disease, and histiocytosis. Therefore, RDD should also be considered in the diagnosis of the mediastinal infiltrative lesion in young patients with a higher FDG uptake.

Although 20–50% of RDD patients are self-limited, there is still a proportion of patients suffering from prolonged disease or multiple organ invasion [15]. At present, surgery remains the primary treatment regimen for RDD, especially in cases with obstructive/compressive symptoms in vital organs. Other treatments, such as corticosteroids, chemotherapy, low-dose interferon, antibiotics, and radiotherapy, are used as complementary strategies, and prognostic evaluation can be achieved using PET/CT.

## 4. Conclusions

Isolated thymus involvement of RDD is rare, although other extranodal involvements are common. Here, we reported a case of primary thymic RDD, which should be considered as a differential diagnosis of a patient who presented with a hypermetabolic anterior mediastinal mass on ^18^F-FDG PET/CT.

## Data Availability

Not applicable.
